# Si-Ni-San Prevents Reserpine-Induced Depression by Inhibiting Inflammation and Regulating CYP450 Enzymatic Activity

**DOI:** 10.3389/fphar.2019.01518

**Published:** 2020-01-17

**Authors:** Yang Zong, Ting Chen, Hongli Dong, Lijing Zhu, Wenzheng Ju

**Affiliations:** ^1^Clinical Pharmacy Laboratory of TCM, Suzhou TCM Hospital Affiliated to Nanjing University of Chinese Medicine, Suzhou, China; ^2^Clinical Pharmacy Laboratory of TCM, Academy of Wumen Chinese Medicine, Suzhou, China; ^3^Department of Clinical Pharmacology Laboratory, Affiliated Hospital to Nanjing University of Chinese Medicine, Nanjing, China

**Keywords:** depression, reserpine, Si-Ni-San, anti-inflammation, CYP450 enzymes

## Abstract

Depression is becoming a major public health concern worldwide. Si-Ni-San (SNS) is a famous formula in Traditional Chinese Medicine (TCM) with potent antidepressant effects. However, the antidepressant mechanism of SNS has not been clearly elucidated. This study was performed to verify whether the antidepressant effects of SNS were related to its anti-inflammatory effects, the levels of brain-derived neurotrophic factor (BDNF) and Cytochrome P450 (CYP450) enzymatic activity. In our study, behavioral tests such as the forced swim test, sucrose preference test and open-field test were evaluated to ensure the establishment of depressive rats. The levels of IL-1β, IL-6, and TNF-α in the serum, liver, and hippocampus of rats were measured by enzyme-linked immunosorbent assays (ELISA). Furthermore, the key proteins NF-κB, BDNF, and TrkB were analyzed by Western blot (WB) analysis in the hippocampus. In addition, CYP450 enzymatic activity analysis was performed using LC-MS/MS in conjunction with drug and statistics (DAS 3.0) after oral administration of six probe drugs. Our results showed that SNS attenuated reserpine-induced increases in IL-1β, IL-6, and TNF-α expression in the serum, liver, and hippocampus. The levels of NF-κB, BDNF, and TrkB in the hippocampus of depressive rats were also altered. According to the pharmacokinetic parameters, SNS had moderate inhibitory effects in the reserpine-induced depression model on CYP1A2, CYP2D1, CYP2E1, and CYP3A2, but no significant metabolic changes to CYP2C6 and CYP2D2. These findings suggested that SNS has a protective effect on reserpine-induced depressive rats, which may be related to the improvement of the inflammatory factors, the level of BDNF and the activity of CYP450 enzymes.

## Highlights

1. SNS decreased IL-1β, IL-6, and TNF-α expression in the serum, liver, and hippocampus of reserpine-induced depressive rats.
2. SNS changed the protein levels of NF-κB, BDNF, and TrkB in the hippocampus of reserpine-induced depressive rats.
3. SNS may treat the depressive rat nervous system by altering CYP450 enzymatic activity in the liver.

## Introduction

Depression is a clinical syndrome characterized by significant and persistent depression, speech loss, mental and motor retardation, and various physical symptoms and dysfunctions (Jiang et al., 2018). Although antidepressants can improve some mental states of depression, these drugs have more side effects, such as apathy, sleep disorders, and cognitive dysfunction (Hsiao et al., 2015; Vu and Shaya, 2017). Traditional Chinese Medicine (TCM), as a complementary and alternative medicine, has been proven to possess satisfactory effects against depression and its clinical complications. When treating depression, TCM starts from the whole body, considering not only the psychological problems that result from a patient's nervous system disorder but also the changes of the zang-fu organs, qi, and blood (Wang et al., 2014). Si-Ni-San (SNS), a famous formula, was first recorded in the “Treatise on Febrile Diseases” by Zhong-jing Zhang, a famous doctor in the Han Dynasty. It comprises *Bupleuri radix* (Chaihu), *Paeoniae radix alba* (Shaoyao), *Aurantii fructus immaturus* (Zhishi), and *Glycyrrhizae radix et Rhizoma* (Gancao) (Hu et al., 2016). Generally, adult patients take orally 100 ml of SNS at the dosage of 0.09 g/ml twice per day according to the suggestion of traditional Chinese physician. SNS can soothe the liver, remove internal heat, strengthen the spleen, and nourish the blood. Furthermore, it has been clinically applied for the improvement of mental diseases, including depression and other conditions (Shen et al., 2016). Although SNS has certain clinical effects in the treatment of depression (Wang et al., 2009), the lack of rigorous experimental research counteracts the unique advantages of TCM, which seriously hinders its promotion and application worldwide.

Recently, clinical and preclinical evidence have suggested that inflammation is associated with the complicated pathophysiology of depression (Kaufmann et al., 2017; Pfau et al., 2018; Chan et al., 2019). Patients with depression exhibited an upregulation of proinflammatory cytokines, including interleukin-1β (IL-1β), IL-6, and tumor necrosis factor-α (TNF-α) (Kennedy et al., 2009). NF-κB has been known to act as an essential transcription factor for the expression of inflammatory mediators, such as iNOS, COX-2, IL-1β, and TNF-α, which leads to depressive-like behavior (Lawrence and Fong, 2010; Biesmans et al., 2013; Yuan et al., 2015). BDNF and its receptor TrkB are widely distributed in the central nervous system, especially in the cerebral cortex and hippocampus, having an important influence on the survival, growth, and differentiation of neurons. Presently, it is generally believed that a decrease in BDNF and TrkB protein levels is related to the occurrence of depression, and antidepressant treatment can improve the levels of BDNF and TrkB (Duman and Monteggia, 2006). Therefore, this study evaluated the efficacy of SNS by detecting these biomarker changes in depressive rats.

The multi-component and multi-target characteristics of TCM determine the complexity of its mechanism of action. The evaluation of the enzymatic activities of the main active ingredients can provide a scientific basis for understanding the rational application and mechanism of TCM (Li et al., 2013). In general, the clinical significance of metabolic interactions due to enzyme inhibition is much greater than that of enzyme promotion, accounting for approximately 70% of all interactions in the enzyme system. The inhibition of CYP450 enzymes by the active ingredient in TCM can reduce the elimination of metabolism of other TCM ingredients or chemical drugs, break the balance between the combined drugs and their metabolites, increase their blood concentration or cause accumulation, and may lead to an enhancement in the drug efficacy. However, adverse reactions or toxic reactions occur. Therefore, evaluating the effect of the active ingredients of TCM on CYP450 enzymatic activity is of great significance for both the clinical safety and reducing the risk of drug-drug interactions (Overholser and Foster, 2011). Cocktail analysis using probe drugs can be used to efficiently study the activity of various CYP enzymes (Lammers et al., 2016). In this study, we described the development and validation of six probe drugs for CYP1A2, CYP2C6, CYP2D1, CYP2D2, CYP2E1, and CYP3A2 using LC-MS/MS to determine CYP450 enzymatic activities in rats. The probe drugs cocktail comprised six representative substrates: theophylline (THE) for CYP1A2, tolbutamide (TOL) for CYP2C6, omeprazole (OME) for CYP2D1, dextromethorphan (DXT) for CYP2D2, chlorzoxazone (CZX) for CYP2E1, and midazolam (MDZ) for CYP3A2.

Therefore, in order to evaluate the curative effect of SNS, biochemical indexes such as proinflammatory cytokines in the hippocampus, serum, and liver tissue were measured in reserpine-induced depressive rats. To elucidate the effect of SNS on CYP450 enzymatic activity, the changes to the six probe substrates in the depression model rats were studied by an LC-MS/MS method. These data can provide sound scientific evidence for the clinical treatment of depression.

## Materials and Methods

### Animals

Male Sprague-Dawley rats weighing 200–220 g (Nantong University, certificate number SCXK-(SU) 2014-0001) were maintained under standard conditions in an animal house (Animal Research Center, Jiangsu Provincial Hospital of TCM, Nanjing). Animals were kept at 22°C –25°C with free access to water and food under a 12:12 h light/dark cycle. The lights were turned on every day at 8:00 a.m. Rats were acclimated to the laboratory for 5–7 days before the experiments. This study was approved and conducted according to the institutional guidelines of the Animal Care and Use Committee at Nanjing University of Chinese Medicine (Nanjing, China).

### Herbs, Chemicals, and Reagents

The herbs used to prepare SNS are *Bupleurum chinense DC*. (Chaihu), *Paeonia lactiflora Pall*. (Shaoyao), *Citrus aurantium L*. (Zhishi), and *Glycyrrhiza uralensis Fisch. ex. DC*. (Gancao). All herbs were provided by the pharmacy of Jiangsu Province Hospital of Chinese Medicine (Nanjing, China) and were authenticated by Prof. Shengjin Liu (Nanjing University of Chinese Medicine). SNS was supplied in the form of a water-extracted cream that was manufactured from a mixture of the crude drugs according to a fixed ratio listed in **Table 1**. Briefly, these 4 dried herbal were soaked in water for 30 min, and then were extracted with boiling water (1:8) twice for 2 h each time. After collecting all the solution, placed in a water bath at 60°C and evaporated to a target concentration for frozen storage. The solutions of the herb preparation and vehicle were administered to rats *via* intragastric administration at a dosage of 10 ml/kg (body weight), and the concentration of the solutions for administration was 0.15 g/ml (medium dose), 0.30g/ml (high dose). The medium dose is the clinical equivalent, the low-dose group was given half the medium dose.

**Table 1:** Crude drug composition of Si-Ni-San.

Plant name	Chinese name	Place of origin	Composition (g)	Major components
*Bupleurum chinense DC*.	Chaihu	Gansu	2.25	Saikosaponin A
*Paeonia lactiflora Pall*.	Shaoyao	Anhui	2.25	Paeoniflorin
*Citrus aurantium L*.	Zhishi	Jiangxi	2.25	Hesperidin Neohesperidin Naringin
*Glycyrrhiza uralensis Fisch. ex. DC*.	Gancao	Inner Mongoria	2.25	Liquiritin

Fluoxetine hydrochloride capsule was purchased from Lilly suzhou pharmaceutical Co. Ltd (Suzhou, China), reserpine injection was purchased from Tianjin jinyao pharmaceutical Co. Ltd (Tianjin, China), midazolam injection and omeprazole enteric-coated capsules were purchased from Changzhou siyao pharmaceutical Co. Ltd (Changzhou, China). Theophylline (CAS No. 58-55-9, purity 99%), tolbutamide (CAS No. 64-77-7, purity 99%), dextromethorphan (CAS No. 125-71-3, purity 98%), and chlorzoxazone (CAS No. 95-25-0, purity 98%) were purchased from Sigma Aldrich (St. Louis, MO, USA); tinidazole (CAS No. 19387-91-8, purity 99%) was purchased from the China Institute of Food and Drug Control (Beijing, China). Reference standards of paeoniflorin (CAS No. 23180-57-6, purity ≥98%), naringin (CAS No. 10236-47-2, purity ≥98%), and hesperidin (CAS No. 520-26-3, purity ≥98%) were purchased from National Institutes for Food and Drug Control (Beijing, China); liquiritin (CAS No. 551-15-5, purity ≥98%), neohesperidin (CAS No. 13241-33-3, purity ≥98%), and saikosaponin A (CAS No. 20736-09-8, purity ≥98%) were purchased from Chengdu munster biotechnology Co., Ltd (Chengdu, China). Acetonitrile (ACN) and methanol (MeOH) were obtained from Merck KGaA (Darmstadt, Germany) and both were of LC-MS grade.

### Determination of Si-Ni-San

Quantitative analysis of the six specific components in the SNS methanol extract was performed on an HPLC (Agilent Technologies 1100 series, USA) with a diode array detector (DAD) detector. Analysis was carried out with a common C18 column (150 mm × 4.6 mm, 5 µm), and the column temperature was maintained at 30°C. The mobile phase was composed of A (water) and B (acetonitrile) using a gradient elution of 15% B from 0–7 min; 15%–17% B from 7–13.5 min; 17%–18% B from 13.5–22.5 min; 18%–30% B from 22.5–28.5 min; 30%–40% B from 28.5–34.5 min; 40%–50% B from 34.5–42.5 min; 50%–80% B from 42.5–45 min; and 80%–15% B from 45–55 min. The flow rate was set to 1 ml/min. Monitoring was performed at 203 nm. The injection volume was 20 μl. Saikosaponin A, paeoniflorin, hesperidin, naringin, neohesperidin, and liquiritin were carefully weighed into a 5 ml volume flask and diluted to scale with methanol for final concentrations of 1.016 mg/ml, 0.946 mg/ml, 1.016 mg/ml, 1.162 mg/ml, 2.16 mg/ml, and 0.970 mg/ml, respectively. Finally, the concentrations of the six compounds from the methanol extract of SNS were calculated by a standard curve method.

### Experimental Design and Drug Administration

Experiment 1: 48 male SD rats were randomly divided into six groups. A control group (no reserpine treatment), a depression model group (reserpine treatment), three SNS groups (reserpine treatment + SNS at concentrations of 0.75, 1.5, and 3.0 g/kg, p.o.), and a venlafaxine group (reserpine treatment + venlafaxine 15 mg/kg, p.o.). The groups were recorded as A, B, C, D, E, and F, respectively.

Experiment 2: 32 male SD rats were randomly divided into four groups. A control group (no reserpine treatment), a depression model group (reserpine treatment), an SNS group (reserpine treatment + SNS 3.0 g/kg, p.o.), and a venlafaxine group (reserpine treatment + venlafaxine 15 mg/kg, p.o.). The groups were recorded as A, C, F, and H, respectively.

**Figure 1:**
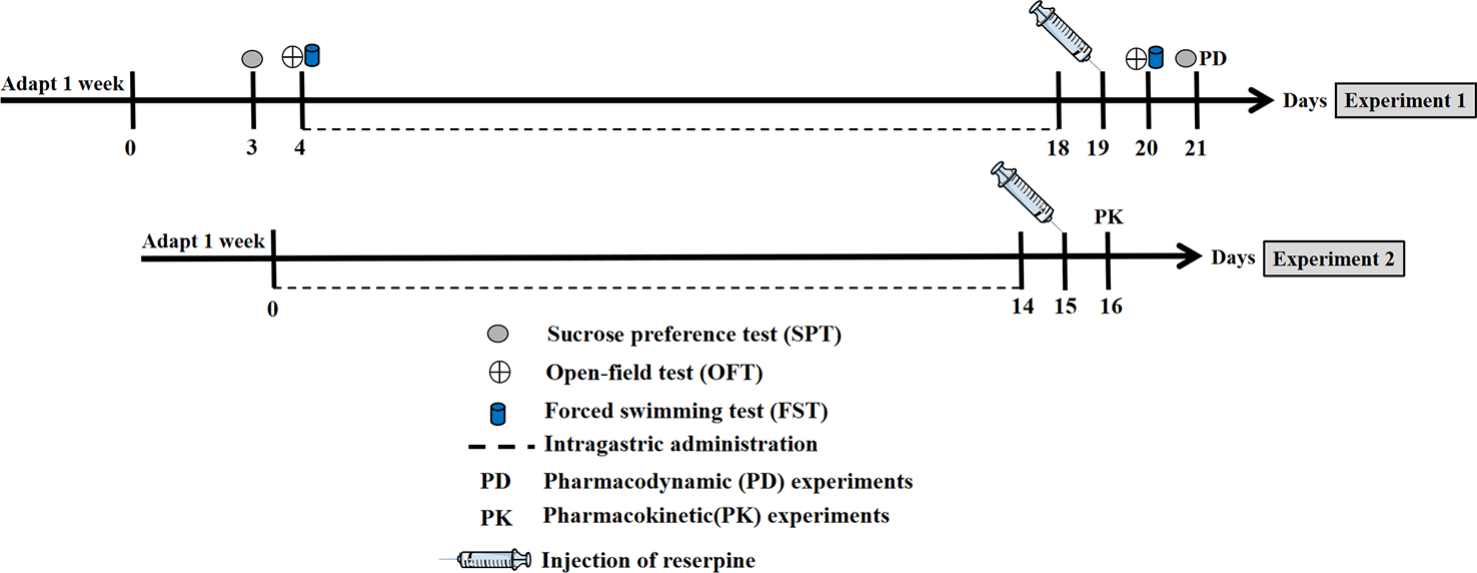
Schematic diagram of the experimental procedures.

After 1 week of adaptation, groups C to F were given their corresponding drugs for 2 weeks, and groups B to F were intraperitoneally injected with 4 mg/kg reserpine on the 19th day while group A was injected with the same volume of normal saline. Experiment 1: The behavioral tests started 2 h after the model had been completed, and the pharmacodynamic (PD) experiments were conducted on the second day. Experiment 2: Each group of rats received cocktail probes orally, and the CYP450vs enzymatic activities were measured 2 h after the model was completed. The experimental design is shown in **Figure 1**.

### Behavioral Tests

#### Weight Change

The weights of the rats in each group were measured weekly, and the growth rate of the rat weights was calculated according to the following equation:

$$\text{Weight change}=(W_{2}-W_{1})/W_{1}$$

where (W_2_-W_1_) is the difference between the weekly weight of the rats, and W_1_ is the weight of the rats in the previous week.

#### Temperature (T/°C)

An infrared noncontact body temperature-measuring instrument was used to measure the temperature of the rats. This method is simple, fast, and reduces the errors from conventional thermometers that stimulate rats, resulting in an increase in temperature.

#### Open-Field Test (OFT)

The locomotor activity of the rats was measured by performing the open field test 48 h after reserpine injection. A 50 × 50 × 50 cm square open carton was made by our team. The white paint pen was divided into 25 small squares of 10 × 10 cm on the inner wall; the paths of the animal were then recorded with a video camera above the arena and analyzed with a video tracking system (DigBehav system, Yishu Co., Ltd.). The inner wall and bottom of the box were cleaned to remove the last remaining odour, which would affect the results of the following tests. The horizontal score, the number of trapping boxes, the vertical score, the number times the upper limbs were lifted and the number of self-modification times were observed in 5 min.

#### Forced Swimming Test (FST)

Rats were allowed to swim for 15 min the day before the FST (Fernandes et al., 2008). On the day of the test, individual rats were separately introduced into two individual cylindrical tanks (65 cm high × 30 cm diameter) with water (25°C), in which the rats cannot touch the bottom of the tank or escape. The total immobility time was measured during the last 4 min of a 6 min trial using video tracking software (SMART 3.0; Panlab S.I., Barcelona, Spain).

#### Sucrose Preference Test (SPT)

The specific methods of selecting 1% sucrose water were as follows, using two bottles: the 1st day, the two bottles were filled with 200 ml of 1% sucrose water; on the 2nd day, 200 ml of 1% sucrose water and 200 ml of pure water were used; on the 3rd day, there was no diet or drinking water; on the 4th day, 200 ml of 1% sucrose water and 200 ml of pure water were used; and on the 5th day, the consumption of sugar water and purified water was measured. The sucrose preference quotiety was calculated according to the following equation:

SPT=S/(S+W)×100%

where S represents the amount of 1% sucrose solution consumed (ml) and W represents the pure water consumed (ml).

### Detection of IL-1β, IL-6, and TNF-α by Enzyme-Linked Immunosorbent Assay (ELISA)

The concentrations of IL-1β, IL-6, and TNF-α in the rat serum, liver, and hippocampus were analyzed by an IL-1β and IL-6 ELISA kit (Yi Fei Xue Biotechnology Co., Ltd., Nanjing, China), and a TNF-α ELISA kit (Cusabio, Wuhan, China). Six duplicate wells were set up for each group.

### Western Blot (WB) Analysis

Total proteins were prepared from the hippocampus, which were lysed using Mammalian Lysis Buffer (Sigma Aldrich), and immunoblotting was performed according to the manufacturer's protocol (Bio-Rad, Hercules, CA). The levels of the target protein bands were determined by using Image J density measurements (NIH, Bethesda, MD).

### Determination of CYP450 Enzymatic Activity

#### Chromatographic Condition

Chromatographic separation was performed on an Agilent Zorbax SB C18 (2.1 mm × 150 mm, 5 μm) column with an Agilent Eclipse XDB2 C8 (2.1 mm × 12.5 mm, 5 μm) Security Guard Cartridge. A gradient program was employed with the mobile phases of solvent A (acetonitrile) and solvent B (0.1% formic acid aqueous solution with 4 mM ammonium formate) at a flow rate of 0.2 ml/min, and the column temperature was maintained at 35°C during the analysis.

#### Mass Spectrometer Conditions

Samples were analyzed using an LC system coupled to Waters Quattro Micro mass spectrometer (Waters, USA) with an electrospray ionization (ESI) source. Positive and negative ions were detected simultaneously. The ESI needle voltage was adjusted to 3.0 kV, and the turbo-gas temperature was set at 350°C. Multiple reaction monitoring (MRM) mode is described in **Table 1**. Data procurement was controlled by MassLynx 4.0.

#### Plasma Sample Preparation

Approximately 300 µl of blood was collected from the eyelids at 0, 5, 10, 20, and 40 min, and 1, 1.5, 2, 2.5, 3, 5, 8, 12, and 24 h after administration of the probe drugs. Each blood sample was centrifuged at 3,000 × g for 10 min at 4°C to obtain 100 µl of plasma. Plasma samples (10 µl) were mixed with 800 µl of ethyl acetate and 20 µl tinidazole (IS). After vortexing for 3 min, the sample was centrifuged at 12,000 rpm for 10 min at 4°C. Then, 750 µl of supernatant was dried under N_2_ at 40°C and dissolved in 100 µl of 75% acetonitrile. One hundred microliters of the supernatant was centrifuged at 12,000 rpm for 5 min at 4°C. Afterwards, 80 µl of the supernatant was transferred to an autosampler vial and 10 µl was injected into the LC-MS/MS system.

#### Establishment of Methodologies

The developed method was validated according to the latest FDA and EMA guidelines for bioanalytical method validation (EMA and CHMP, 2011; U.S. Food and Drug Administration, 2018).

#### Linearity

The linearity of the method was assessed by rat plasma with increasing amounts of THE, TOL, OME, DXT, CZX, and MDZ. Twenty microliters of the mixed reference series solution and 20 μl of the internal standard were added to 100 μl of blank plasma. The plasma samples were treated using the rat plasma sample treatment method. The ratio of the peak area (Ai) of each component to the corresponding internal standard peak area (As) was calculated as Y. The linear regression of the probe drug concentration X was carried out with the ratio Y, and the regression equation was obtained.

#### Precision and Accuracy

To investigate the accuracy and precision, 100 µl of blank plasma was added to 20 µl of the low, medium and high concentrations of THE, TOL, OME, DXT, CZX, and MDZ. The low concentrations of the six probe drugs were 0.92, 0.46, 1.14, 0.42, 0.55, and 0.39 μg/ml; the medium concentrations of the six probe drugs were 7.40, 3.66, 9.12, 3.34, 4.40, 3.12 μg/ml; and the high concentrations (quality control) of the six probe drugs were 59.20, 29.28, 72.98, 26.70, 35.18, 31.44 μg/ml, respectively. The samples were treated according to method 2.6.4. Five microliters of each sample was used for analysis. The detection values of each analyte were calculated according to the corresponding line. The results were repeated 5 times in one day and 3 days in parallel. Matrix effects were addressed by preparing six quality control sets in processed blank matrix as well as preparing a diluted stock solution and subsequently comparing the resulting peak area ratios (analyte/internal standard).

#### Stability

The stability assessment covered the short-term, post-preparative, and freeze-thaw stability, and six quality control sets were used in each case. For the short-term stability, samples were stored at room temperature for 1 h prior to treatment. Post-preparative stability was tested by placing the final samples in an autosampler at 4°C for 12 and 24 h. Samples underwent up to three freeze-thaw cycles before they were processed. The results were compared to the results of samples analyzed immediately. Stability was assumed if the drug content after the given storage condition was within the acceptable range of accuracy, i.e., ± 15%.

### Statistical Analyses

All data in the present research are expressed as the mean ± SD, and the statistical analyses were performed using GraphPad Prism 6.0 (GraphPad Software Inc., San Diego, CA, USA). The data from the behavioral tests, ELISA, and WB were analyzed using one-way analysis of variance (ANOVA) followed by *post hoc* Tukey's multiple comparison test. A non-compartmental model was used to evaluate the PK parameters of the substrates and metabolites using DAS 3.0 (version 3.0, Scientific Consulting, KY, USA). A value of *P <* 0.05 was considered to be statistically significant for analysis.

## Results

### Quantitative Analysis of Components in SNS

**Figure 2:**
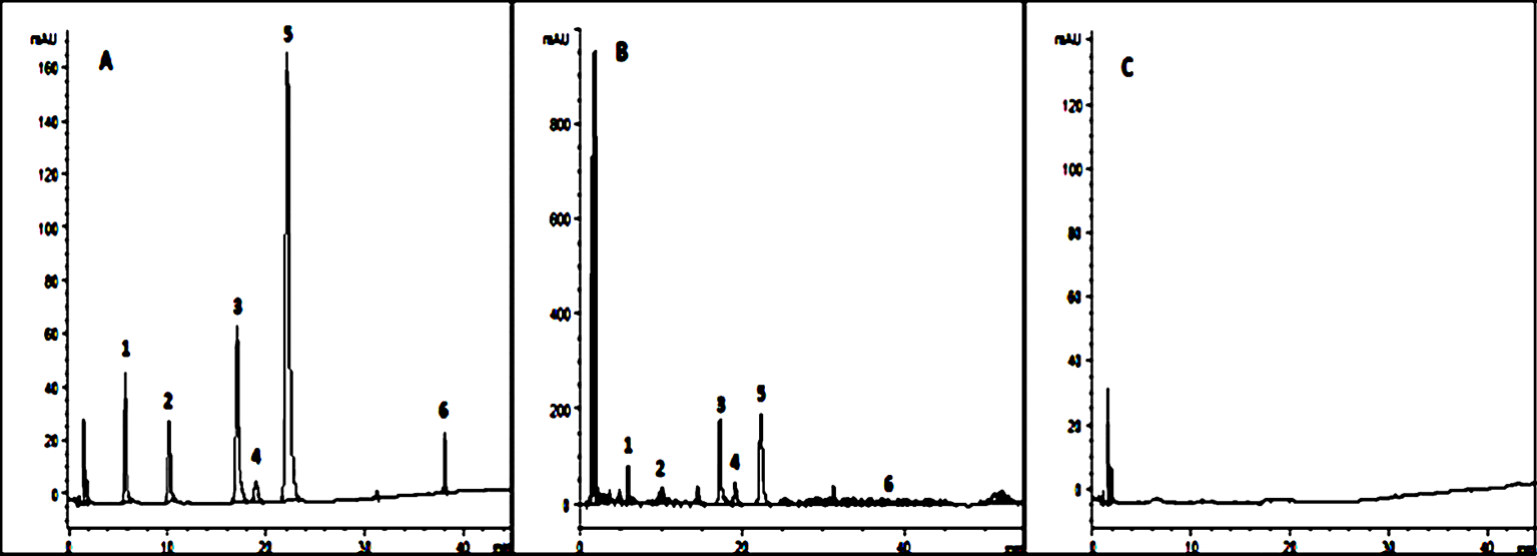
Six kinds of ingredients in SNS chromatograms. **(A)** Reference substance solution; **(B)** Sample solution; **(C)** Methanol solution; 1. Paeoniflorin, 2. Liquiritin, 3. Naringin, 4. Hesperidin, 5. Neohesperidin, 6. Saikosaponin A.

In order to ensure the quality of the prescription form of TCM, we chose six major compounds from SNS for quantitative analysis by HPLC-DAD (**Figure 2**) according to the Pharmacopoeia of the People's Republic of China (2015 version). They were 10.72 mg/g saikosaponin A, 7.11 mg/g paeoniflorin, 3.09 mg/g hesperidin, 14.20 mg/g naringin, 11.84 mg/g neohesperidin, and 0.68 mg/g liquiritin in the methanol extract of SNS.

### Validation of the Depression Model

Depression-like behavior evaluated by the OFT, FST, and SPT is shown in **Table 2**. The horizontal and vertical score in the OFT was used to estimate the rat's motor function and state anxiety, and the immobility time in the FST was used to assess the degree of despair in rats. The evaluation parameters had a minor nuance between the two groups before depression model establishment but significantly varied after model establishment (*P <* 0.01). The horizontal and vertical scores significantly decreased from 103.88 ± 12.48 to 56.34 ± 12.33 and from 17.63 ± 2.00 to 5.34 ± 3.12 (*P <* 0.01) in the model group, respectively. After the model was established, significant decreases were seen for the scores between the two groups (*P <* 0.01). The immobility time increased from 74.50 ± 6.63 to 102.47 ± 6.8 within the model group (*P <* 0.01). A notable difference was also observed between the two groups at the end of model establishment (*P <* 0.01).

**Table 2:** The data of behavioral tests in reserpine-induced depressive rats (mean ± SD, *n* = 8).

Time	Behavior test		Control	Model
Before modeling	Weight change		0.165 ± 0.0245	0.167 ± 0.0273
	T/°C		36.33 ± 0.30	36.21 ± 0.31
	OFT	Horizontal score	108.67 ± 13.65	103.88 ± 12.48
		Vertical score	21.33 ± 2.12	17.63 ± 2.00
	FST/s		71.17 ± 9.33	74.50 ± 6.63
	SPT/%		92.34 ± 3.82	91.23 ± 2.35
Modeling 2 weeks	Weight change		0.138 ± 0.0264	0.145 ± 0.0245
	T/°C		36.43 ± 0.10	36.46 ± 0.28
	OFT	Horizontal score	102.34 ± 11.34	56.34 ± 12.33^ΔΔ^
		Vertical score	20.13 ± 2.34	5.34 ± 3.12^ΔΔ^
	FST/s		73.21 ± 9.23	102.47 ± 6.86^ΔΔ^
	SPT/%		90.23 ± 3.23	64.45 ± 2.43^ΔΔ^

^ΔΔ^P < 0.01 in comparison with control group.

The sucrose preference quotiety of the rats was used to reflect the rat's reactivity to reward. Significant changes occurred for SPT in the model group from 91.23 ± 2.35 to 64.45 ± 2.43 (*P <* 0.01). There were obvious alterations in the SPT after the model was built (*P <* 0.01).

### Effects of SNS on IL-1β, IL-6, and TNF-α Levels in the Rat Serum, Liver, and Hippocampus

The effects of SNS on inflammatory cytokines are shown in **Table 3**. The reserpine-induced rat depression model significantly elevated the expression levels of IL-1β, IL-6, and TNF-*α* in the serum, liver and hippocampus of rats compared with the control group (*P* < 0.01 or *P* < 0.05). In contrast to the SNS (low dose) group, where there was no significant difference in liver IL-6 (*P >* 0.05), all other SNS dose groups and the venlafaxine group levels of IL-1β, IL-6, and TNF-*α* were significantly lower than the model group (*P <* 0.01 or *P <* 0.05). These data indicate that SNS may help regulate the immune and endocrine dysfunction associated with depression.

**Table 3:** Expression of IL-1β, IL-6, and TNF-α in hippocampus, liver, and serum.

Parameters	IL-1β (ng/L)	IL-6 (ng/L)	TNF-α (ng/L)
**Hippocampus**			
A	16.69 ± 0.93**	45.19 ± 3.17**	17.12 ± 0.97**
B	46.73 ± 3.23^##^	153.77 ± 4.04^##^	102.78 ± 2.90^##^
C	30.21 ± 1.00**^##^	93.04 ± 2.31**^##^	66.37 ± 3.31**^##^
D	24.17 ± 1.87**^##^	81.40 ± 0.23**^##^	71.10 ± 4.67**^##^
E	14.98 ± 0.58**	67.23 ± 3.77**^#^	42.59 ± 1.95**^##^
F	17.12 ± 1.23**	51.74 ± 3.08**	47.56 ± 1.88**^##^
**Liver**			
A	22.50 ± 1.05*	39.61 ± 2.03**	70.97 ± 3.73
B	26.16 ± 1.11^#^	53.79 ± 2.29^##^	71.71 ± 3.90
C	16.41 ± 0.86**^#^	56.90 ± 1.98^##^	51.13 ± 3.90**^##^
D	23.18 ± 1.72*	45.38 ± 3.68*	55.76 ± 3.90*^#^
E	18.12 ± 0.44**^#^	34.25 ± 1.30**^#^	52.77 ± 4.67*^#^
F	22.36 ± 1.19*	44.46 ± 3.18*	61.47 ± 0.97*^#^
**Serum**			
A	13.50 ± 0.92**	27.59 ± 1.58**	30.19 ± 2.78**
B	38.06 ± 2.50^##^	199.39 ± 7.33^##^	103.02 ± 3.05^##^
C	28.16 ± 1.13**^##^	115.60 ± 5.67**^##^	52.11 ± 3.56**^##^
D	23.69 ± 1.92**^##^	105.38 ± 3.30**^##^	54.08 ± 0.69**^##^
E	12.98 ± 0.44**	57.77 ± 1.64**^#^	50.22 ± 4.15**^##^
F	12.08 ± 0.91**	53.59 ± 1.00**^#^	30.24 ± 2.70**

Data are presented as mean ± SD (n = 8), compared with control group, ^#^P < 0.05, ^##^P < 0.01; compared with model group B, *P < 0.05, **P < 0.01. A: control group; B: model group; C, low dose group of SNS; D: middle dose group of SNS; E: high dose group of SNS; F: venlafaxine group.

### Effects of SNS on NF-κb, BDNF, and TrkB Level in the Rat Hippocampus

**Figure 3:**
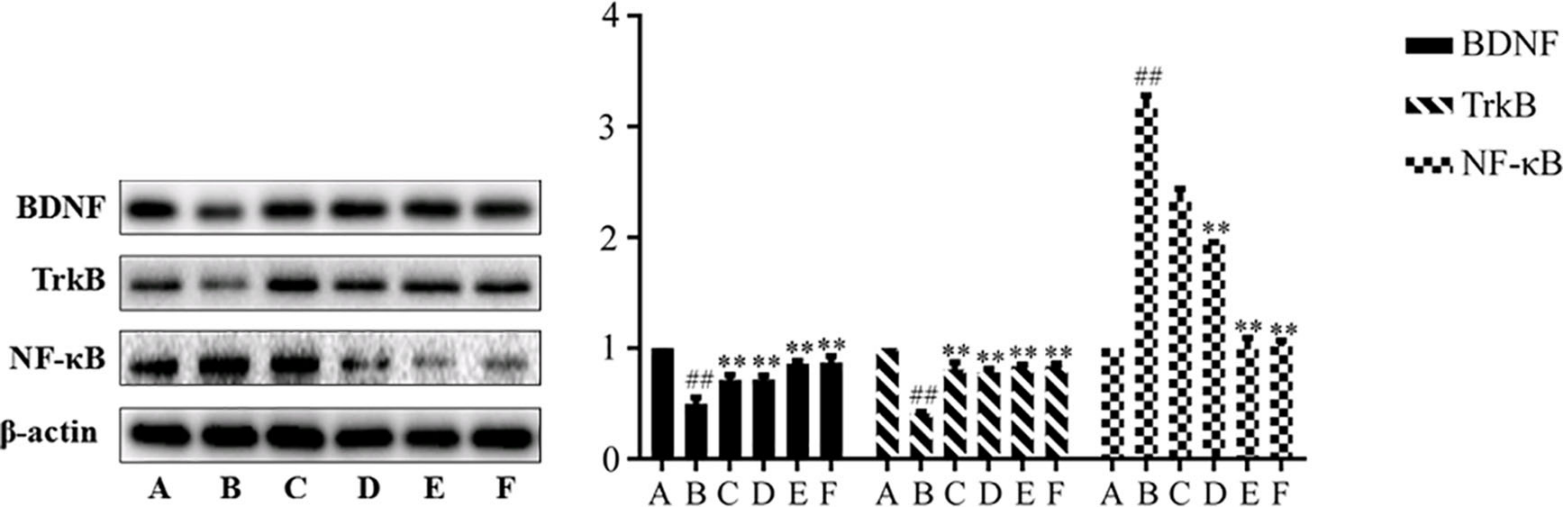
Expression of BDNF, TrkB, and NF-κB in hippocampus. Data are presented as mean± SD (*n* = 8), compared with control group, ^##S^*P* < 0.01; compared with model group B, ^##^*P* < 0.01. (A) Control group; (B) Model group; (C) Low dose group of SNS; (D) Middle dose group of Si-Ni-San (SNS); (E) High dose group of SNS; (F) Venlafaxine group.

The effects of SNS on NF-κB and BDNF and its receptor TrkB are presented in **Figure 3**. The reserpine-induced rat depression model significantly expressed higher levels of NF-κB, BDNF, and TrkB in the hippocampus of rats compared with the control group (*P* < 0.01). After drug intervention, the levels of NF-κB, BDNF, and TrkB were significantly improved (*P* < 0.01), which suggests that SNS can affect hippocampal neuronal activity in reserpine-induced depressive rats.

### Effects of SNS on CYP450 Enzymatic Activity

**Figure 4:**
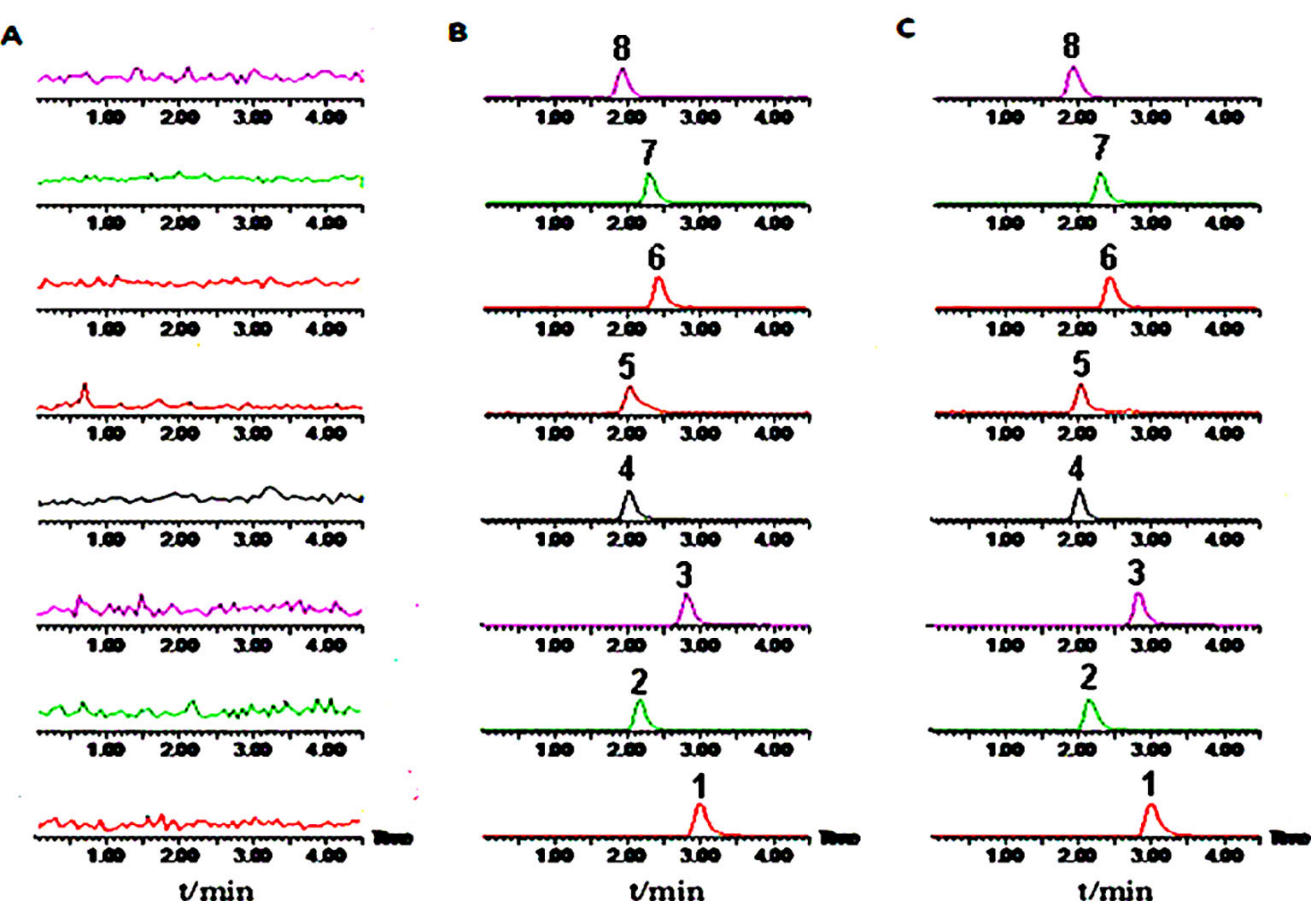
Representative multiple reaction monitoring (MRM) chromatograms of six probe drugs and internal standards. **(A)** Blank plasma; **(B)** Plasma added with reference standards; **(C)** Plasma samples. 1, Midazolam; 2, Omeprazole; 3, Dextromethorphan; 4, Tinisazole (IS+); 5, Tinisazole (IS-); 6, Tolbutamide; 7, Chlorzoxazone; 8, Theophylline.

The plasma concentrations of the six probe drugs were simultaneously determined by LC-MS/MS after oral administration. After investigating the ions produced by collision-induced dissociation, the m/z transitions selected for MRM analysis were determined and optimized for each compound (**Table 4**). The MRM chromatograms of the six substrates are shown in **Figure 4**. No endogenous source of interference was observed. All probe drugs tested in the present cocktail were evaluated for matrix effects (**Table 5**), linearity (**Table 6**), extraction recovery (**Table 5**), precision (**Table 7**), accuracy (**Table 7**), and stability (**Table 8**). These results indicated that the developed method was applicable for the simultaneous quantitative analysis of substrates in rat plasma samples.

**Table 4:** Mass spectrometry parameters for detection in the multiple reaction monitoring mode.

Probes	Polarity	Precursor (m/z)	Product (m/z)	DP (V)	CE (eV)
THE	ESI-	179.3	164.3	30	20
CZX	ESI-	168.2	168.1	30	5
TOL	ESI-	269.3	170.2	20	20
TXZ(IS)	ESI-	246.5	246.4	25	5
DXT	ESI+	272.1	171.1	25	35
OME	ESI+	346.0	198.0	10	10
MDZ	ESI+	326.1	291.2	35	32
TXZ(IS)	ESI+	248.5	248.4	30	5

Dwell time was in each case 20 ms. Mass to charge ratio (m/z) transitions printed in italic were used for quantification. Theophylline, THE; Chlorzoxazone, CZX; Tolbutamide, TOL; Tinisazole (IS-), TXZ; Dextromethorphan, DXT; Omeprazole, OME; Midazolam, MDZ; CE, collision energy; DP, declustering potential.

**Table 5:** Recovery and matrix effect of six probe drugs and IS. Results are expressed as mean ± SD, n = 3.

Probes	Concentration (μg/ml)	Recovery($\bar{x}$ ± SD)/%	Matrix effect ($\bar{x}$ ± SD)/%
	0.925	64.28 ± 1.24	87.55 ± 0.88
THE	7.400	52.89 ± 4.46	89.01 ± 1.63
	59.20	70.12 ± 0.61	90.31 ± 2.47
	0.457	84.44 ± 3.25	92.96 ± 4.01
CZX	3.659	76.64 ± 6.02	90.75 ± 1.34
	29.28	85.11 ± 7.96	96.41 ± 2.29
	1.140	45.06 ± 9.34	91.86 ± 5.70
TOL	9.122	48.98 ± 6.41	97.28 ± 7.30
	72.98	50.79 ± 9.24	95.40 ± 3.38
	0.417	40.38 ± 7.67	102.23 ± 6.87
DXT	3.338	50.51 ± 6.05	91.52 ± 5.07
	26.70	56.48 ± 5.71	90.10 ± 3.96
	0.550	54.73 ± 7.56	104.58 ± 9.03
OME	4.397	57.85 ± 9.10	99.91 ± 4.10
	35.18	60.68 ± 4.70	100.40 ± 1.23
	0.391	63.99 ± 1.12	87.14 ± 0.51
MDZ	3.125	62.63 ± 8.19	88.35 ± 4.05
	31.44	69.08 ± 2.48	91.42 ± 1.28
TXZ(+)	13.60	65.77 ± 4.10	92.84 ± 2.08
TXZ(−)	13.60	90.95 ± 7.86	102.44 ± 8.50

**Table 6:** Standard curves of six probe drugs.

Proubes	Calibration equation	Calibration range (μg/ml)	R^2^	LLOQ (μg/ml)
THE	*Y* = 1.782*X* + 1.849	0.155 ~ 188.40	0.9974	0.155
CZX	*Y* = 45.22*X* + 20.21	0.0767 ~ 46.84	0.9986	0.0767
TOL	*Y* = 5.020 *X* + 7.579	0.191 ~ 291.90	0.9990	0.191
DXT	*Y* = 0.0219 *X* + 0.0034	0.0700 ~ 42.72	0.9963	0.0700
OME	*Y* = 0.0269*X* + 0.0127	0.0922 ~ 56.28	0.9958	0.0922
MDZ	*Y* = 0.0338 *X* + 0.0118	0.0655 ~ 40.00	0.9960	0.0655

**Table 7:** Summary of intra-day (3 replicates per concentration) and inter-day (three individual runs) precision and accuracy of quality control samples for THE, CZX, TOL, DXT, OME, MDZ in rat plasma. Results are expressed as mean ± SD; RSD, relative standard deviation.

Proubes	Concentration (μg/ml)	Inter-day (*n* = 5)	Precision (RSD)/%	Intra-day (*n* = 15)	Precision (RSD)/%
		Accuracy ($\bar{x}$ ± SD)/%		Accuracy ($\bar{x}$ ± SD)/%	
	0.925	100.96 ± 3.01	2.99	101.99 ± 5.28	5.18
THE	7.400	101.39 ± 2.91	2.87	101.14 ± 5.44	5.38
	59.20	102.27 ± 3.71	3.63	100.45 ± 3.97	3.96
	0.457	100.33 ± 9.21	9.18	100.90 ± 7.23	7.20
CZX	3.659	98.62 ± 6.16	6.24	96.54 ± 6.46	6.69
	29.28	101.28 ± 7.79	7.69	100.64 ± 4.72	4.69
	1.140	93.66 ± 6.55	6.99	96.95 ± 7.38	7.61
TOL	9.122	96.73 ± 4.63	4.78	100.92 ± 5.64	5.59
	72.98	101.59 ± 5.59	5.50	100.92 ± 3.94	3.90
DXT	0.417	101.52 ± 7.41	7.30	100.86 ± 5.36	5.31
	3.338	105.08 ± 7.77	7.39	99.47 ± 7.43	7.47
	26.70	95.59 ± 6.44	6.73	99.29 ± 5.96	6.00
	0.550	106.87 ± 6.60	6.18	101.23 ± 6.51	6.42
OME	4.397	102.58 ± 7.36	7.18	103.99 ± 5.62	5.40
	35.18	97.72 ± 3.97	4.07	100.16 ± 4.88	4.87
	0.391	95.28 ± 5.84	6.13	100.02 ± 8.10	8.10
MDZ	3.125	106.77 ± 4.95	4.63	102.48 ± 5.34	5.22
	31.44	103.72 ± 3.52	3.39	101.49 ± 4.36	4.29

**Table 8:** Short-term stability (1 h), rack stability (24 h), and freeze-thaw stability (3rd cycle) stability results for THE, CZX, TOL, DXT, OME, MDZ in rat plasma. Results are expressed as mean ± SD, n = 3.

Probes	Concentration (μg/ml)	Short-term stability 1 h/%	Rack stability 24 h/%	Freeze-thaw stability 3rd cycle/%
	0.925	94.24 ± 3.24	95.65 ± 2.51	98.85 ± 4.32
THE	7.400	95.54 ± 2.61	97.14 ± 4.73	94.55 ± 5.12
	59.20	97.13 ± 4.21	97.13 ± 3.65	101.33 ± 4.24
	0.457	98.13 ± 2.15	101.29 ± 3.54	99.35 ± 4.52
CZX	3.659	101.13 ± 2.25	104.75 ± 4.35	105.35 ± 5.78
	29.28	96.52 ± 3.55	112.43 ± 2.12	114.54 ± 6.14
	1.140	95.15 ± 2.84	94.37 ± 3.12	96.21 ± 4.33
TOL	9.122	94.17 ± 3.24	96.45 ± 2.85	99.85 ± 5.25
	72.98	95.28 ± 2.33	97.55 ± 4.23	102.85 ± 5.14
	0.417	96.57 ± 3.45	95.42 ± 2.25	98.33 ± 4.56
DXT	3.338	102.14 ± 2.25	103.21 ± 4.02	102.25 ± 5.35
	26.70	98.78 ± 3.16	101.22 ± 4.16	106.36 ± 4.85
	0.550	95.17 ± 3.91	100.21 ± 3.44	103.87 ± 5.34
OME	4.397	104.15 ± 4.13	101.35 ± 4.52	106.24 ± 5.97
	35.18	98.25 ± 3.75	104.32 ± 3.75	110.72 ± 6.02
	0.391	94.18 ± 3.14	96.32 ± 2.12	99.24 ± 4.38
MDZ	3.125	96.85 ± 2.35	99.24 ± 3.42	103.45 ± 5.78
	31.44	102.28 ± 3.74	104.85 ± 4.15	108.76 ± 6.24

**Figure 5:**
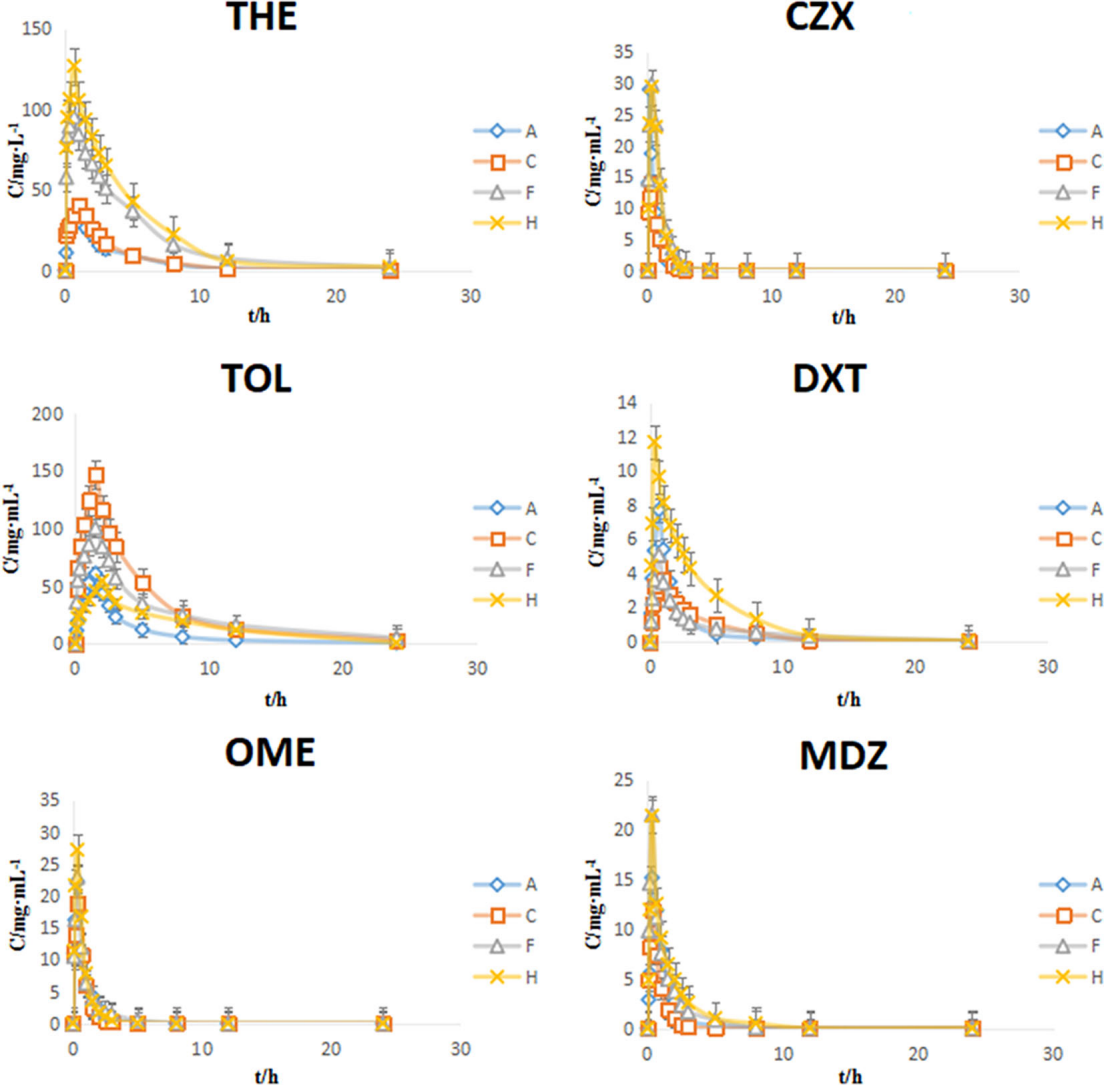
Plasma concentration-time curves of six probe drugs in rats (mean ± SD, n = 8). A: control group, C: model group, F: high dose group of Si-Ni-San (SNS), H: venlafaxine group.

The PK parameters of the substrates and the concentration-time curve are presented in **Table 9** and **Figure 5**, respectively. According to the FDA evaluation and classification of inhibitors and inducers (Huang et al., 2007), the main pharmacokinetic parameters of THE, OME, and CZX in the model group showed no significant change when compared with the control group (*P* > 0.05). However, the values of *AUC*_(0-t)_ and *C*_max_ for TOL in the model group were 2.65-fold and 2.43-fold higher than those of the normal group, respectively; the values of *AUC*_(0-t)_ and *C*_max_ for DXT in the model group were 2.03-fold higher and 1.78-fold lower than those of the normal group, respectively; and the values of *AUC*_(0-t)_ and *C*_max_ for MDZ in the model group were 2.70-fold and 1.40-fold lower than those of the normal group (*P* < 0.01 or *P* < 0.05). The results indicate that the reserpine-induced depression model has a moderate inhibitory effect on CYP2D1 and CYP2D2 but and a moderate inducing effect on CYP3A2.

**Table 9:** Pharmacokinetic parameters of probe drugs (THE, CZX, TOL, DXT, OME, MDZ) in plasma following an oral administration at a dose of 10 mg/kg probe drugs in rats (mean ± SD, n = 8), compared with the control group, *P < 0.05, **P < 0.01; compared with the model group, ^Δ^*P*< 0.05, ^ΔΔ^*P* < 0.01.

Parameters	A	C	F	H
THE-CYP1A2				
V_d_/F/L	0.0028 ± 0.0005	0.33 ± 0.29^*^	0.0091 ± 0.012^Δ^	0.028 ± 0.034^Δ^
*T_1/2_*/h	2.47 ± 0.88	2.04 ± 0.47	2.58 ± 0.94	2.17 ± 0.75
*T_max_*/h	0.83 ± 0.71	0.90 ± 0.71	0.56 ± 0.17	0.92 ± 0.70
*C_max_*/μg·ml^-1^	34.40 ± 11.65	40.19 ± 15.05	95.06 ± 20.71^ΔΔ^	126.30 ± 14.26^ΔΔ^
AUC_0-t_/μg·ml^-1^·h	201.81 ± 55.68	222.34 ± 83.97	587.93 ± 143.91^ΔΔ^	1084.13 ± 120.57^ΔΔ^
AUC_0-∞_/μg·ml^-1^·h	197.94 ± 52.50	222.58 ± 84.06	588.58 ± 199.88^ΔΔ^	1124.74 ± 187.46^ΔΔ^
AUMC_0-t_/μg·ml^-1^·h^2^	510.16 ± 93.73	859.23 ± 340.15*	2287.24 ± 929.17^ΔΔ^	2600.89 ± 221.67^ΔΔ^
AUMC_0-∞_/μg·ml^-1^·h^2^	574.02 ± 41.73	865.61 ± 343.97	2358.35 ± 856.32^ΔΔ^	2663.44 ± 161.53^ΔΔ^
CZX-CYP2E1				
V_d_/F/L	0.286 ± 0.13	3.18 ± 0.70**	0.015 ± 0.016^ΔΔ^	0.045 ± 0.047^ΔΔ^
*T_1/2_*/h	0.46 ± 0.30	0.41 ± 0.12	0.34 ± 0.22	0.39 ± 0.25
*T_max_*/h	0.22 ± 0.063	0.22 ± 0.083	0.33 ± 0.10	0.28 ± 0.13
*C_max_*/μg·ml^-1^	29.79 ± 8.64	14.23 ± 2.40**	29.08 ± 6.83^ΔΔ^	28.56 ± 12.95^Δ^
AUC_0-t_/μg·ml^-1^·h	8.05 ± 2.58	10.53 ± 3.12	32.00 ± 14.56^ΔΔ^	28.15 ± 12.95^ΔΔ^
AUC_0-∞_/μg·ml^-1^·h	9.15 ± 3.01	10.46 ± 3.19	32.16 ± 14.56^ΔΔ^	28.27 ± 12.93^ΔΔ^
AUMC_0-t_/μg·ml^-1^·h^2^	3.69 ± 1.40	5.56 ± 2.13	26.86 ± 20.77^Δ^	22.68 ± 13.09^Δ^
AUMC_0-∞_/μg·ml^-1^·h^2^	4.58 ± 0.99	5.86 ± 1.97	27.52 ± 21.03^Δ^	23.08 ± 13.04^Δ^
TOL-CYP2C6				
V_d_/F/L	0.73 ± 0.15	0.24 ± 0.30**	0.79 ± 0.10^ΔΔ^	0.31 ± 0.35^ΔΔ^
*T_1/2_*/h	3.76 ± 1.12	5.17 ± 2.02	8.06 ± 2.43^Δ^	5.81 ± 1.46^Δ^
*T_max_*/h	1.60 ± 0.42	1.83 ± 0.26	1.33 ± 0.60	2.17 ± 1.21
*C_max_*/μg·ml^-1^	60.04 ± 25.62	146.35 ± 45.87**	100.57 ± 35.33	54.33 ± 35.52
AUC_0-t_/μg·ml^-1^·h	631.38 ± 248.71	1673.19 ± 479.02**	1368.07 ± 459.69	1324.61 ± 347.10
AUC_0-∞_/μg·ml^-1^·h	664.32 ± 274.06	1790.73 ± 448.16**	1448.82 ± 360.97	1383.85 ± 333.06
AUMC_0-t_/μg·ml^-1^·h^2^	4469.01 ± 1751.26	13329.74 ± 3111.09**	12861.64 ± 5041.97	9778.16 ± 1571.68
AUMC_0-∞_/μg·ml^-1^·h^2^	5307.17 ± 2278.45	16859.02 ± 2478.24**	13046.46 ± 5298.17	9778.16 ± 1502.84
DXT-CYP2D2				
V_d_/F/L	29.84 ± 14.00	29.79 ± 4.30	46.24 ± 9.12^ΔΔ^	5.29 ± 1.82^ΔΔ^
*T_1/2_*/h	1.62 ± 0.57	11.36 ± 2.67**	2.90 ± 1.28^ΔΔ^	2.14 ± 1.48^ΔΔ^
*T_max_*/h	0.63 ± 0.34	0.58 ± 0.47	0.53 ± 0.49	0.33 ± 0.12
*C_max_*/μg·ml^-1^	7.69 ± 2.60	4.31 ± 1.21*	5.08 ± 2.09	11.61 ± 3.82^ΔΔ^
AUC_0-t_/μg·ml^-1^·h	17.10 ± 6.55	34.66 ± 3.18**	29.30 ± 5.93	33.71 ± 3.64
AUC_0-∞_/μg·ml^-1^·h	17.46 ± 6.73	33.28 ± 2.40**	29.69 ± 5.66	35.16 ± 4.72
AUMC_0-t_/μg·ml^-1^·h^2^	18.41 ± 7.83	331.26 ± 15.22**	229.19 ± 27.34^ΔΔ^	253.06 ± 26.95^ΔΔ^
AUMC_0-∞_/μg·ml^-1^·h^2^	19.85 ± 7.10	363.02 ± 26.95**	233.74 ± 28.78^ΔΔ^	253.69 ± 25.48^ΔΔ^
OME-CYP2D1				
V_d_/F/L	0.12 ± 0.036	1.90 ± 0.74**	0.58 ± 0.35^ΔΔ^	6.70 ± 1.54
*T_1/2_*/h	0.291 ± 0.0828	0.68 ± 0.24**	0.77 ± 0.61	0.48 ± 0.13
*T_max_*/h	0.23 ± 0.088	0.25 ± 0.088	0.17 ± 0.092	0.25 ± 0.14
*C_max_*/μg·ml^-1^	22.06 ± 6.65	18.85 ± 8.78	22.02 ± 6.87	27.12 ± 10.58
AUC_0-t_/μg·ml^-1^·h	20.13 ± 10.45	13.93 ± 5.91	27.94 ± 7.95^ΔΔ^	29.43 ± 16.48^Δ^
AUC_0-∞_/μg·ml^-1^·h	20.60 ± 10.46	14.20 ± 5.90	29.06 ± 19.26^ΔΔ^	30.06 ± 16.30^Δ^
AUMC_0-t_/μg·ml^-1^·h^2^	18.44 ± 10.03	11.85 ± 6.78	40.72 ± 23.68^Δ^	24.62 ± 9.16^Δ^
AUMC_0-∞_/μg·ml^-1^·h^2^	18.30 ± 9.75	13.77 ± 8.32	39.89 ± 20.33^Δ^	21.16 ± 10.20^Δ^
MDZ-CYP3A2				
V_d_/F/L	0.12 ± 0.088	0.33 ± 0.24	4.66 ± 1.46^ΔΔ^	0.20 ± 0.17
*T_1/2_*/h	0.81 ± 0.66	0.44 ± 0.17	0.68 ± 0.15^Δ^	0.87 ± 0.29^Δ^
*T_max_*/h	0.29 ± 0.16	0.26 ± 0.15	0.21 ± 0.10	0.25 ± 0.14
*C_max_*/μg·ml^-1^	15.93 ± 1.79	11.35 ± 3.51*	21.56 ± 12.11	21.48 ± 6.66^ΔΔ^
AUC_0-t_/μg·ml^-1^·h	24.81 ± 16.27	9.11 ± 2.02*	25.12 ± 14.75^Δ^	51.16 ± 8.39^ΔΔ^
AUC_0-∞_/μg·ml^-1^·h	27.69 ± 17.35	9.22 ± 2.05*	25.81 ± 15.00^Δ^	53.98 ± 12.30^ΔΔ^
AUMC_0-t_/μg·ml^-1^·h^2^	28.67 ± 13.70	9.13 ± 1.56	30.88 ± 19.27^Δ^	56.37 ± 13.72^ΔΔ^
AUMC_0-∞_/μg·ml^-1^·h^2^	30.77 ± 14.29	9.10 ± 2.68	30.90 ± 17.10^Δ^	55.88 ± 11.68^ΔΔ^

A: control group, C: model group, F: high dose group of SNS, H: venlafaxine group.

Compared with the model group, the values of *AUC*_(0-t)_ and *C*_max_ for THE, CZX, OME, and MDZ in the SNS and venlafaxine groups showed significant changes (*P* < 0.01 or *P* < 0.05); the values of *AUC*_(0-t)_ and *C*_max_ for TOL and DXT in the SNS and venlafaxine groups showed no significant changes (*P* > 0.05). Our results indicate that the SNS and venlafaxine groups have a moderate inhibitory effect on CYP1A2, CYP2D1, CYP2E1, CYP3A2 and no effect on CYP2C6, CYP2D2.

## Discussion

Since the 1950s, reserpine has been used to treat hypertension, which is a central sedative, and a patient who used reserpine in the long-term would have a depression (Leith and Barrett, 1980). Reserpine is a vesicle reuptake inhibitor that leaves the capsule outside of the vesicle, causing behavioral and physiological changes. Antagonism of the behavior and physiological changes caused by reserpine was the first developed depression animal model. Animals treated with reserpine showed sagging, decreased body temperature, and tonic symptoms (Antkiewicz-Michaluk et al., 2014), which were treated with tricyclic antidepressants and monoamine oxidase inhibitors and can antagonize the symptoms of upper eye sagging and body temperature decrease. Studies have found that reserpine is widely used in the modeling of depression, Parkinson's disease and schizophrenia (Fernandes et al., 2012; Park et al., 2018). The reserpine-induced depression rat model can be divided into two types, one for a low dose (i.p., 0.1–0.5 mg/kg) and one for a single high dose (i.p., 1–5 mg/kg) (Fernandes et al., 2012; Ozerov et al., 2016). According to the pre-experiment, a single dose of 4.0 mg/kg reserpine induced depressive symptoms in model rats and was significantly more effective than a single dose of 0.3 mg/kg of reserpine; for example, to decrease the exercise activity in the OFT, to increase the exercise time in the FST and to reduce the sugar water preference in the SPT. Based on the criteria of the behavior evaluation, the induced depression model of reserpine (4.0 mg/kg) was established, and the experimental basis was provided for the follow-up experiment.

Cytokines can be divided into inflammatory cytokines, including IL-1β, IL-6, TNF-α, and TNF-β, and anti-inflammatory cytokines, including IL-4, IL-10, and IL-13. Among them, IL-1β, IL-6, and TNF-α are the key cytokines that initiate the inflammatory response, so they are also called proinflammatory cytokines (Calleja-Castillo et al., 2013). NF-κB can stimulate the expression of cytokines such as TNF-α and IL-1β. These cytokines are resistant to NF-κB, further stimulate NF-κB and aggravate the occurrence of inflammatory reactions (Zhu et al., 2014). In 2006, Duman RS proposed the hypothesis of neurotrophic factors in depression, suggesting that BDNF can promote the growth of sudden contact and maintain the survival of neurons. A decrease in BDNF may lead to brain dysfunction and induce depression. Increasing the level of BDNF in the brain can play a role in the treatment of depression (Duman and Monteggia, 2006). Since then, interest in the role of BDNF and its receptor TrkB has increased. BDNF and its receptor TrkB have high levels of expression in the epidermis in the hippocampus and participate in many kinds of regulation, such as emotions, behavior, learning, and memory. The combination of BDNF and TrkB can activate the neurotrophic factor pathway and repair injured hippocampal neurons (Chen et al., 2013; Serra et al., 2017). Our experiments showed that SNS decreased IL-1β, IL-6, and TNF-α expression in the serum, liver, and hippocampus of depressive rats induced by reserpine. SNS changed the protein levels of NF-κB, BDNF, and TrkB in the hippocampus after oral administration of SNS. The results of the pharmacodynamics experiments demonstrated that the effect of high-dose SNS on reserpine-induced depressive rats is comparable to venlafaxine. Therefore, only the high-dose SNS group was selected to evaluate the effects of SNS on CYP450 enzymes.

The cocktail probe method was first proposed by Breimer (Breimer et al., 1990); that is, a variety of relatively low doses of the probe substrate are administered simultaneously, and the pharmacokinetic parameters of each of the probe substrates in the biological sample are determined to obtain phenotypic information on the plurality of the metabolizing enzymes. The cocktail probe method has the advantage of being simple and rapid, and is mostly currently used in animal or liver microsomes to study CYP450 enzymes. The present study developed and validated a method for the simultaneous quantification of six CYP450 probe drugs in particular in rat plasma: THE (CYP1A2), TOL (CYP2C6), OME (CYP2D1), DXT (CYP2D2), CZX (CYP2E1), and MDZ (CYP3A2). From the pharmacokinetic parameters, it can be seen that after the administration of SNS and venlafaxine, the concentration (C*_max_*) of theophylline, clobamate, ormeiramine, and prochloronil *in vivo* in rats significantly increased and its metabolism slowed down, indicating that the SNS had a strong inhibitory effect on the metabolism of CYP1A2, CYP2D1, CYP2E1, CYP3A2, and had no effect on CYP2C6, CYP2D2. It can be estimated that SNS may have a drug-like effect by interfering with the CYP450 enzymatic activity to treat depression. This also provides a theoretical basis for clinical rational administration.

**Figure 6:**
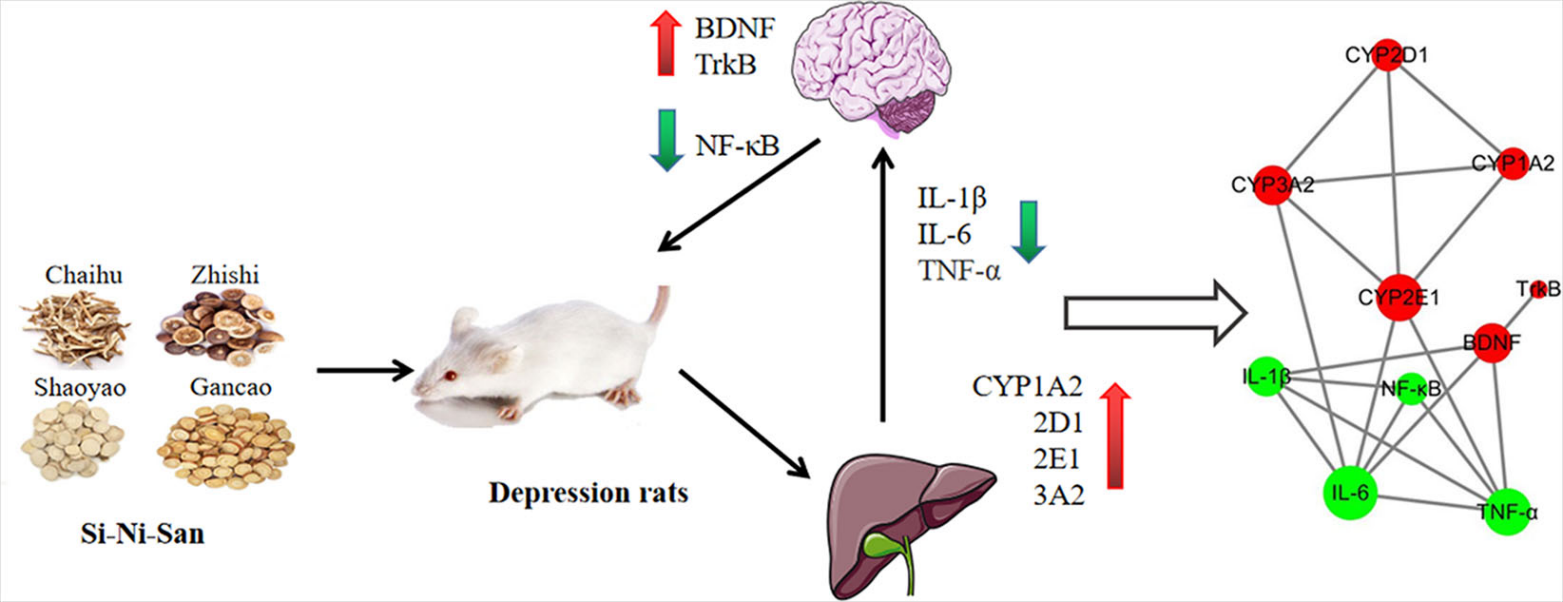
Mechanism of Si-Ni-San (SNS) on the treatment of depressive rats nervous system in the brain by changing the activity of CYP450 enzymes in the liver.

Finally, we used the STRING11.0 database (Szklarczyk et al., 2015) to predict the relationship between CYP450 enzymes and inflammation index. It was found that SNS may up-regulate CYP1A2, CYP2D1, CYP2E1, and CYP3A2 activity to reduce IL-6 and TNF-α expression, as well as up-regulation of BDNF and its receptor TrkB, reduce IL-1β, IL-6, and TNF-α expression to treat depression (**Figure 6**).

## Conclusion

These results indicated that SNS can improve reserpine-induced indicators of depression. The pharmacodynamic mechanism may down-regulate the inhibition of activation of inflammatory cytokines (IL-1β, IL-6, TNF-α, and NF-κB) and the improvement of BDNF and TrkB in the hippocampus in depressive rats caused by reserpine. The results of the pharmacokinetic parameters show that SNS has a clear effect on the depressive symptoms and the metabolic enzymes induced by reserpine. We predict that the underlying mechanism of SNS treatment of depression may be through the rat cerebral nervous system by altering the activity of CYP450 enzymes in the liver (**Figure 6**).

## Data Availability Statement

The datasets generated for this study are available on request to the corresponding author.

## Ethics Statement

The animal study adhered to the institutional guidelines of the Animal Care and Use Committee at Nanjing University of Chinese Medicine.

## Author Contributions

WJ designed the experiment. LZ performed the animal operation and tissue collection. HD supervised the research and conducted the statistical analyses. YZ and TC drafted and revised the manuscript, and all authors approved the manuscript.

## Funding

The authors disclosed receipt of the following financial support for the research, authorship, and/or publication of this article: this study was supported by grants from the National Natural Science Foundation of China (81573685), the Suzhou Science and Technology Bureau Guiding Topic (SYSD2019149), and the Hospital-level project of Suzhou TCM Hospital (YQN2017004, YQN20108022).

## Conflict of Interest

The authors declare that the research was conducted in the absence of any commercial or financial relationships that could be construed as a potential conflict of interest.
